# Past and Future of Analog-Digital Modulation of Synaptic Transmission

**DOI:** 10.3389/fncel.2019.00160

**Published:** 2019-04-24

**Authors:** Mickael Zbili, Dominique Debanne

**Affiliations:** ^1^UNIS, UMR 1072, INSERM AMU, Marseille, France; ^2^CRNL, INSERM U1028—CNRS UMR5292—Université Claude Bernard Lyon1, Lyon, France

**Keywords:** axon, ion channels, synaptic transmission, brain circuits, short-term plasticity

## Abstract

Action potentials (APs) are generally produced in response to complex summation of excitatory and inhibitory synaptic inputs. While it is usually considered as a digital event, both the amplitude and width of the AP are significantly impacted by the context of its emission. In particular, the analog variations in subthreshold membrane potential determine the spike waveform and subsequently affect synaptic strength, leading to the so-called analog-digital modulation of synaptic transmission. We review here the numerous evidence suggesting context-dependent modulation of spike waveform, the discovery analog-digital modulation of synaptic transmission in invertebrates and its recent validation in mammals. We discuss the potential roles of analog-digital transmission in the physiology of neural networks.

## The Action Potential Is Not a Digital Event

**Figure 1:**
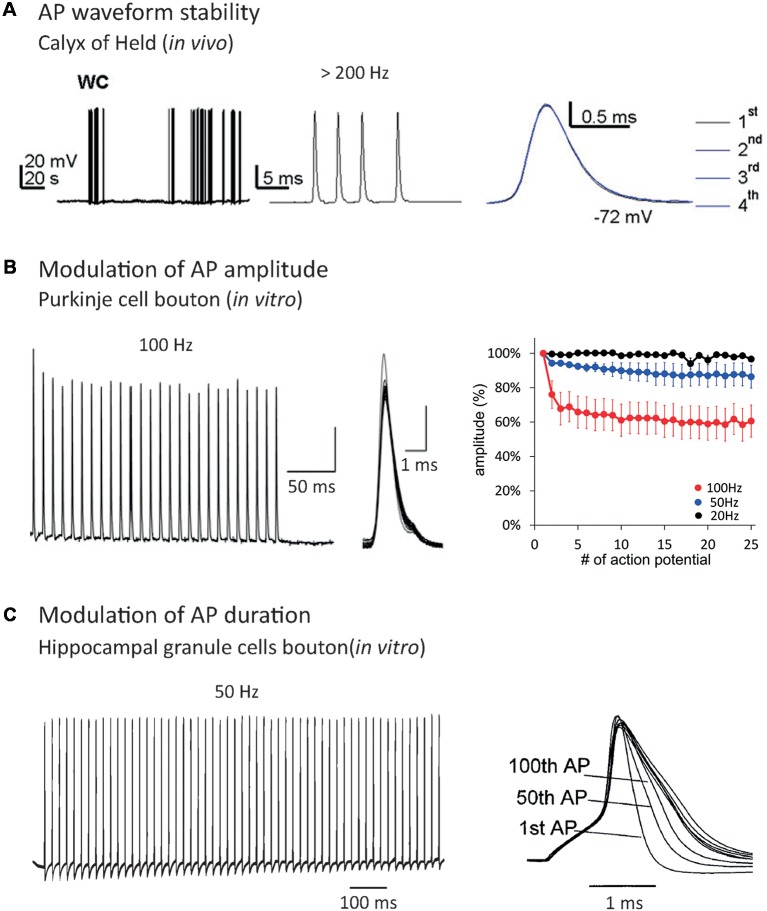
Effect of repetitive firing on axonal Action Potential (AP) shape. **(A)** AP waveform is highly stable during high-frequency trains in the Calyx of Held recorded *in vivo*. Note that the APs are indistinguishable when they are superimposed. Adapted with permission from Sierksma and Borst (2017). **(B)** AP amplitude decrease during repetitive firing in Purkinje cells bouton. Note that increasing the frequency of AP train provokes an enhancement of AP amplitude decrease. Adapted with permission from Kawaguchi and Sakaba (2015). **(C)** AP duration increase during repetitive firing in hippocampal mossy fiber bouton. Adapted with permission from Geiger and Jonas (2000).

In the central nervous system (CNS), synaptic transmission is mainly supported by APs, i.e., it occurs when a spike has been emitted in the presynaptic cell. Classically, the analogy is made between the spike and the basic unit of information used in computers (bit), i.e., the spike is thought to be the minimal unit of information that a neuron can emit. In this view, the spike is seen as an “all-or-none” digital phenomenon whose shape is constant or whose shape modifications are not relevant for neuronal processing (Maley, 2018). These two assertions are wrong in most of the neuronal cell types, despite some cases showing very stable spike shape (Sierksma and Borst, 2017; Figure 1A).

In most neurons, the spike waveform is highly variable in function of the quantity of voltage-gated channels available at spike emission. This quantity depends on two parameters: the density and the level of inactivation of the channels. In this review, we will focus on variations in axonal spike shape that impact neurotransmitter release and synaptic strength.

The first source of spike shape modification is the neuronal firing rate. Repetitive firing may cause inactivation of both voltage-gated sodium channels (Nav) and voltage-gated potassium channels (Kv). Nav inactivation leads to a decrease in spike amplitude during AP trains (Brody and Yue, 2000; Prakriya and Mennerick, 2000; He et al., 2002; Kawaguchi and Sakaba, 2015; Ma et al., 2017; Ohura and Kamiya, 2018; Figure 1B), while Kv inactivation leads to an increase in spike width (Jackson et al., 1991; Park and Dunlap, 1998; Shao et al., 1999; Geiger and Jonas, 2000; Faber and Sah, 2003; Kim et al., 2005; Deng et al., 2013; Liu et al., 2017; Ma et al., 2017; Figure 1C). When it invades the presynaptic terminal, the spike provokes the opening of voltage-gated calcium channels (Cav), leading to an increase of Ca^2+^ concentration in the bouton and the release of neurotransmitters. Due to the power law between intra-terminal Ca^2+^ concentration and neurotransmitter release, small variations in presynaptic calcium entry, occurring through spike shape modifications, can lead to large changes in synaptic transmission (Sabatini and Regehr, 1997; Bollmann et al., 2000; Bischofberger et al., 2002; Fedchyshyn and Wang, 2005; Yang and Wang, 2006; Bucurenciu et al., 2008; Scott et al., 2008; Neishabouri and Faisal, 2014). In fact, spike broadening during repetitive firing entails synaptic transmission facilitation in the pituitary nerve (Jackson et al., 1991), dorsal root ganglion (Park and Dunlap, 1998) and mossy fiber bouton (Geiger and Jonas, 2000). Other studies showed that spike amplitude depression during repetitive firing provokes a decrease in synaptic transmission at hippocampal (Brody and Yue, 2000; Prakriya and Mennerick, 2000; He et al., 2002) and cerebellar synapses (Kawaguchi and Sakaba, 2015). Therefore, spike shape variations participate in short-term synaptic plasticity produced by repetitive firing (Zucker and Regehr, 2002). In addition, Hebbian or homeostatic forms of synaptic plasticity may result from modulation of presynaptic spike waveform *via* long-term regulation of ion channel density (Gandhi and Matzel, 2000; Yang and Wang, 2006; Hoppa et al., 2014).

Another source of spike waveform variation is the presence of neuromodulators. Neuromodulation alters spike shape *via* subthreshold modifications of membrane potential or channel biophysics regulation. In hippocampal neurons, glutamate and GABA have been shown to depolarize axonal membrane potential leading to spike broadening, probably through Kv channel inactivation, inducing an increase in synaptic transmission (Ruiz et al., 2010; Sasaki et al., 2011). In cortical pyramidal neurons, dopamine fixation on D1 receptors causes a decrease in Kv1-dependent I_D_ current, due to the hyperpolarization of its inactivation curve, leading to axonal spike broadening (Dong and White, 2003; Yang et al., 2013).

Action Potential (AP) waveform in neuronal compartments depends on the local density of voltage-gated ion channels. For example, the AP duration decreases during its axonal propagation in L5 pyramidal neurons due to axonal expression of Kv1 channels (Kole et al., 2007). Recent studies have shown that the density of voltage-gated channels is not homogenous all along the axon, leading to local variation of spike shape during its propagation. The density of peri-terminal Kv3 channels determines local spike width and synaptic release in terminals of cerebellar stellate cell interneurons (Rowan et al., 2016). In axons of cortical neurons, varying density of Nav channels determines branch-specific spike amplitude and spike-evoked presynaptic Ca^2+^ entry (Cho et al., 2017). Therefore, the distribution of synaptic strength in neuronal networks is likely to be in part determined by the variability of AP waveform in the presynaptic terminals.

Finally, spike broadening and increased synaptic release due to Kv channel dysfunction or Kv channel down-regulation has been associated with various neurologic disorders such as schizophrenia, episodic ataxia type 1, fragile X syndrome, autism and epilepsy (Deng et al., 2013; Begum et al., 2016; Crabtree et al., 2017; Vivekananda et al., 2017; Scott et al., 2019).

Therefore, the spike waveform can be modified by neuronal firing rate, neuromodulation, variation in local voltage-gated channel density, voltage-gated channel long-term regulation and dysfunction of voltage-gated channels in the pathological context. All these spike waveform variations modify Ca^2+^ entry and synaptic release at presynaptic terminals. As spike shape modifications alter the transmission of synaptic information, it should not be considered as a purely digital event.

In the following sections, we will focus on spike shape modulation by subthreshold variations of membrane potential. We will see that the spike waveform is determined by an analog information, the subthreshold neuronal activity, leading to synaptic release modulation. This phenomenon has been called Analog-Digital synaptic transmission (Clark and Häusser, 2006; Alle and Geiger, 2008; Debanne et al., 2013; Rama et al., 2015b; Zbili et al., 2016).

## Birth of Analog-Digital Modulation of Synaptic Transmission at Invertebrate Synapses

### AP Amplitude-Dependent Modulation of Synaptic Strength

The modulation of spike-evoked synaptic transmission by modulation of the presynaptic AP waveform has been first reported at the squid giant synapse by Hagiwara and Tasaki (1958). This pioneering study revealed that the amplitude of the presynaptic spike directly determines the amplitude of the postsynaptic response. The EPSP modulation was found to be extremely large, from virtually no detectable response to ~7–8 mV (Hagiwara and Tasaki, 1958). The modulation of the presynaptic spike amplitude was simply obtained by varying the intensity of subthreshold depolarization. This spike-amplitude dependent modulation of synaptic transmission has been confirmed by subsequent studies at the squid giant synapse (Takeuchi and Takeuchi, 1962; Miledi and Slater, 1966; Kusano et al., 1967).

**Figure 2:**
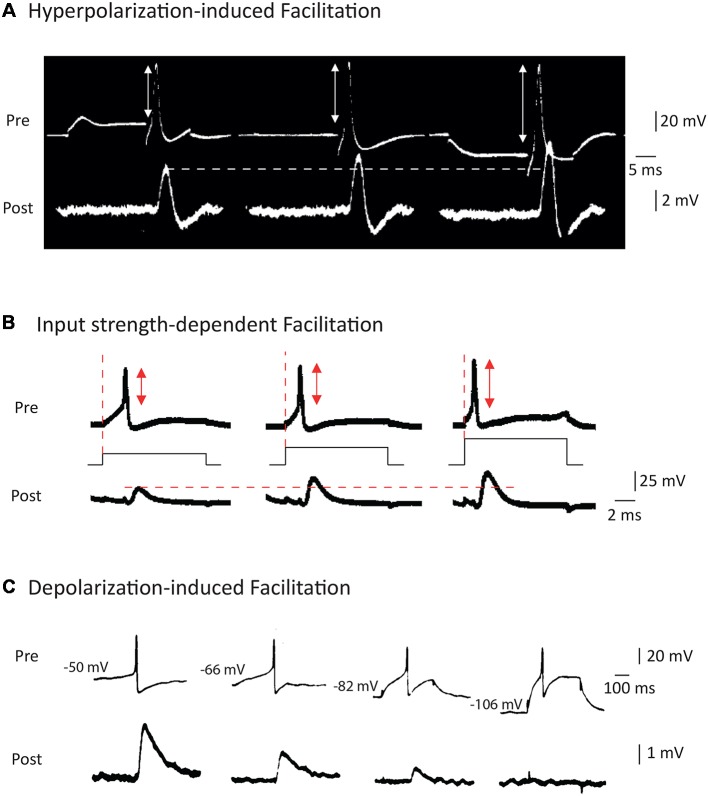
Modulation of AP waveform and synaptic strength by presynaptic membrane potential in invertebrates. **(A)** Hyperpolarization of the presynaptic element leads to an increase in the spike amplitude and the post-synaptic potential amplitude at squid giant synapse. Adapted with permission from Takeuchi and Takeuchi (1962). **(B)** Increasing the current applied to emit the spike leads to a decrease in presynaptic spike latency, an increase in spike amplitude and an increased EPSP amplitude at the squid giant synapse. Adapted with permission from Kusano et al. (1967). **(C)** Depolarization of the presynaptic cell leads to an increase in spike-evoked synaptic transmission at the cholinergic synapse of Aplysia. Adapted with permission from Shapiro et al. (1980).

While it has been suspected for a long time that a presynaptic hyperpolarization increased spike-evoked synaptic transmission (Del Castillo and Katz, 1954), the study by Takeuchi and Takeuchi (1962) represents the first report to unambiguously show that transient hyperpolarization of the presynaptic membrane potential during induction of the presynaptic AP enhanced the presynaptic spike amplitude and, subsequently, the postsynaptic response (Figure 2A). Although the precise mechanisms underlying this modulation have not been addressed in this study, the facilitation may result from recovery of sodium current from inactivation (see also Rama et al., 2015a). This principle has been confirmed in many studies published later, including one in the squid (Miledi and Slater, 1966) and another in the crayfish (Dudel, 1971). Interestingly, this hyperpolarization-induced facilitation of synaptic transmission has also been reported at the rat neuromuscular junction (Hubbard and Willis, 1962, 1968).

The study by Kusano et al. (1967) was largely inspired by the work of Hagiwara and Tasaki (1958) but it represents the first clear demonstration that increasing the input current applied to trigger a spike leads to an increase in both the presynaptic spike amplitude and the postsynaptic response (Figure 2B). These two early studies are somehow without descendants since no other study has been undertaken since. Furthermore, the mechanism of this modulation has never been clearly identified but it may result from the minimization of sodium channel inactivation prior to spike emission.

### AP Duration-Dependent Modulation of Synaptic Strength

Modulation of synaptic strength by AP duration was reported later, after the discovery of enhancement of synaptic transmission by AP amplitude modulation. The first clear study stating context-dependent enhancement of synaptic transmission due to the broadening of presynaptic AP is that of Shapiro et al. (1980). The authors reported that in connected neurons from Aplysia, depolarization of the presynaptic neuron inactivates a potassium current, leading to a broadening of the spike and an enhancement of synaptic transmission (Shapiro et al., 1980; Figure 2C). One should note that this effect is exactly the opposite of that described in the squid where a presynaptic depolarization reduced spike-evoked transmission. Before this study, Shimahara and Tauc (1975) reported similar findings but the mechanism was not studied in this first report (Shimahara and Tauc, 1975). Later, Shimahara confirmed that blocking Kv channels with 4-aminopyridine suppressed the increase in synaptic transmission induced by depolarization (Shimahara, 1981, 1983). However, in these studies, it was unclear whether voltage was mainly acting on spike amplitude or spike duration.

### Presynaptic Voltage-Dependent Modulation: Role of Calcium Current

Beyond inactivation of Kv channels, a second mechanism had been identified in the Shapiro et al.’s (1980) study showing depolarization-induced enhancement of synaptic transmission. They showed that subthreshold depolarization of the presynaptic neuron to −55/−35 mV activated a steady-state Ca^2+^ current that also contributed to the modulation of transmission, possibly by controlling release probability (Shapiro et al., 1980; Connor et al., 1986). Similar depolarization-induced enhancement of synaptic transmission has been reported at an inhibitory synapse in the leech (Nicholls and Wallace, 1978). This study reports that small presynaptic depolarizations increase synaptic strength in an AP waveform independent way (probably due to basal Ca^2+^ accumulation), while stronger depolarizations enhance synaptic release *via* broadening of the presynaptic spike. Therefore, depolarization-induced enhancement of synaptic transmission *via* basal Ca^2+^ increase and *via* spike broadening can coexist at invertebrates’ synapses. Recently, the depolarization-induced enhancement of synaptic transmission *via* basal Ca^2+^ accumulation has been confirmed in heart interneurons of the leech (Ivanov and Calabrese, 2003) and B21 sensory neurons of the Aplysia (Ludwar et al., 2009, 2017; Evans et al., 2011).

Two main features should be noted in these pioneering studies. First, the modulation of synaptic transmission was found to be extremely large (about an order of magnitude). Second, only one type of modulation was found in a given presynaptic cell-type (i.e., only depolarization-induced facilitation in the Aplysia or hyperpolarization-induced facilitation in the squid).

## Recent Developments

From the early studies on spike-evoked release modulation *via* presynaptic membrane potential, we can conclude that spikes contain more information than usually thought. In fact, the synaptic strength depends on the subthreshold membrane potential of the presynaptic cell, indicating that the presynaptic spike transmits this analog information to the postsynaptic cell. However, the direction of this modulation of synaptic transmission seems to depend on the type of synapse. In fact, in some studies, the rule is: the more depolarized is the presynaptic cell, the bigger is the PSP (also called depolarization-induced Analog-Digital Facilitation or d-ADF), while in others the rule is the opposite (hyperpolarization-induced Analog-Digital Facilitation or h-ADF). We will see that the recent developments on the subject have extended the observations made on invertebrate preparations to mammalian synapses and have resolved this apparent paradox *via* the description of the ion channels responsible for the two types of ADF.

### Depolarization-Induced Analog-Digital Facilitation (d-ADF) in Mammalian Brain

**Figure 3:**
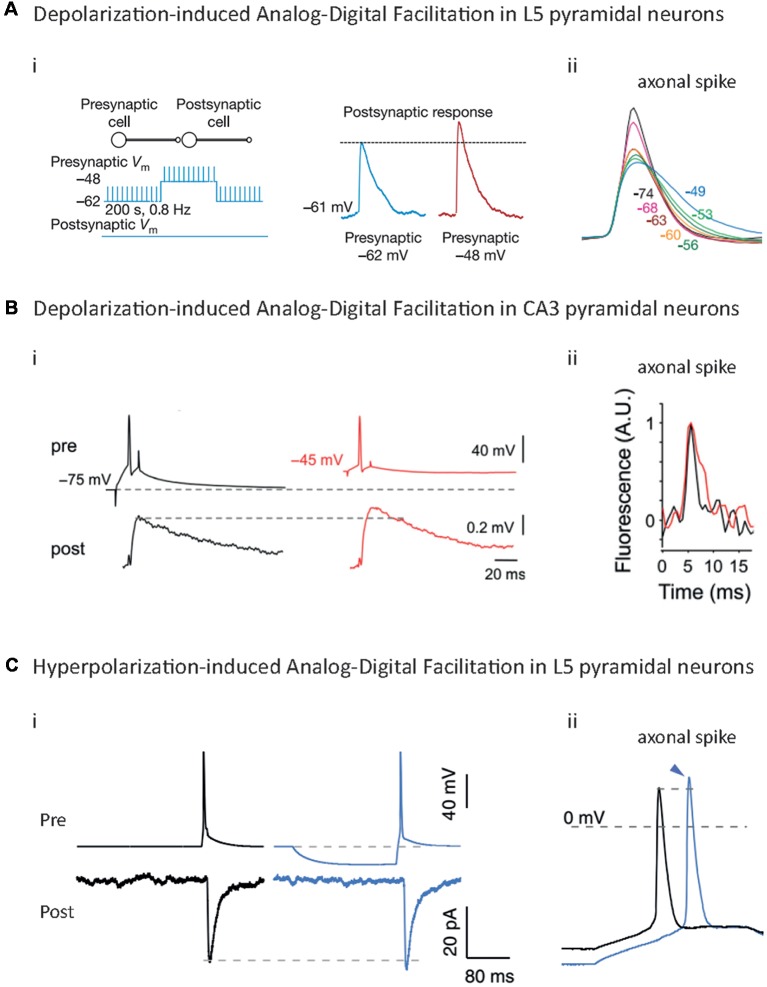
Analog-Digital Facilitations at mammalian synapses. **(A)** Depolarization-induced Analog-Digital Facilitation (d-ADF) at L5-L5 synapses. Depolarization of the presynaptic cell leads to an increase in synaptic transmission at L5/L5 synapses (i) that is due to the broadening of the axonal spike measured by whole-cell recording from an axonal bleb (ii). Adapted with permission from Shu et al. (2006). **(B)** d-ADF at CA3/CA3 synapses. Long depolarization of the presynaptic cell leads to an increase in the synaptic transmission (i) that is mediated by the broadening of the axonal spike measured in voltage imaging (ii). Adapted with permission from Bialowas et al. (2015). **(C)** Hyperpolarization-induced Analog-Digital facilitation (h-ADF). Hyperpolarization of the presynaptic cell leads to an enhancement of synaptic transmission at L5/L5 synapses: (i) due to an increase in the spike amplitude measured by whole-cell recording from an axonal bleb, (ii) adapted with permission from Rama et al. (2015a).

The first descriptions of d-ADF in mammalian CNS have been made in Calyx of Held (Turecek and Trussell, 2001; Awatramani et al., 2005), CA3 area (Saviane et al., 2003), hippocampal mossy fibers (Alle and Geiger, 2006) and L5 pyramidal neurons (Shu et al., 2006; Figures 3Ai,Bi; Table 1). In all these cases, a depolarization of the presynaptic cell preceding the AP leads to an increase in synaptic transmission from 30% to 100% depending on the studies. However, a precise examination shows that different mechanisms are responsible for these d-ADFs.

**Table 1:** Analog-digital facilitation.

	Authors	Species	Cell type	Mechanism
**d-ADF**	Shimahara and Tauc (1975)	Aplysia	Interneuron	Not studied
	Nicholls and Wallace (1978)	Leech	Heart interneuron	Basal Ca^2+^ Kv inactivation AP broadening
	Shimahara and Peretz (1978)	Aplysia	Interneuron	Not studied
	Alle and Geiger (2006)	Rat	Mossy fiber giant bouton	Unknown
	Scott et al. (2008)	Rat	Mossy fiber giant bouton	Unknown
	Zorrilla de San Martin et al. (2017)	Rat	Purkinje cells	Unknown
	Shapiro et al. (1980)	Aplysia	Cholinergic interneuron L10	Basal Ca^2+^ Kv inactivation AP broadening
	Shimahara (1981); Shimahara (1983)	Aplysia	Left pleural ganglion	Kv inactivation AP amplitude increase?
	Saviane et al. (2003)	Rat	CA3 pyramidal neuron	Kv inactivation AP broadening
	Shu et al. (2006)/Shu et al. (2007)	Ferret/Rat	L5 pyramidal neuron	Kv inactivation AP broadening
	Kole et al. (2007)	Rat	L5 pyramidal neuron	Kv inactivation AP broadening
	Ruiz et al. (2010)	Rat	Mossy fiber giant bouton	• AP broadening
	Zhu et al. (2011)	Rat	L5 pyramidal neuron/interneuron synapses	• Kv inactivation
	Sasaki et al. (2011)	Rat	CA3 pyramidal neuron	Kv inactivation AP broadening
	Sasaki et al. (2012)	Rat	CA3 pyramidal neuron	Kv inactivation AP broadening
	Kim (2014)	Rat	CA1 pyramidal neuron/interneuron synapses	Kv inactivation AP broadening
	Bialowas et al. (2015)	Rat	CA3 pyramidal neuron	Basal Ca^2+^ Kv inactivation AP broadening
	Rowan and Christie (2017)	Mouse	Cerebellar interneuron (stellate cell)	Kv inactivation AP broadening
	Connor et al. (1986)	Aplysia	Cholinergic interneuron L10	• Basal Ca^2+^
	Turecek and Trussell (2001)	Rat	Calyx of Held	• Basal Ca^2+^
	Ivanov and Calabrese (2003)	Leech	Heart interneuron	• Basal Ca^2+^
	Ludwar et al. (2009)	Aplysia	Sensory neuron B21	• Basal Ca^2+^
	Evans et al. (2011)	Aplysia	Sensory neuron B21	• Basal Ca^2+^
	Ludwar et al. (2017)	Aplysia	Sensory neuron B21	• Basal Ca^2+^
	Awatramani et al. (2005)	Rat	Calyx of Held	• Basal Ca^2+^
	Hori and Takahashi (2009)	Mouse/Rat	Calyx of Held	• Basal Ca^2+^
	Christie et al. (2011)	Rat	Cerebellar interneuron (Molecular Layer)	• Basal Ca^2+^
	Bouhours et al. (2011)	Rat	Cerebellar interneuron (Molecular Layer)	• Basal Ca^2+^
**h-ADF**	Del Castillo and Katz (1954)	Frog	Neuromuscular junction	Unknown
	Takeuchi and Takeuchi (1962)	Squid	Giant synapse	• AP amplitude increase
	Miledi and Slater (1966)	Squid	Stellate ganglion	• AP amplitude increase
	Dudel (1971)	Crayfish	Motor nerve	• AP amplitude increase
	Hubbard and Willis (1968)	Rat	Neuromuscular junction	• AP amplitude increase
	Hubbard and Willis (1962)	Rat	Neuromuscular junction	• AP amplitude increase
	Thio and Yamada (2004)	Rat	Hippocampal neurons	Unknown
	Cowan and Stricker (2004)	Rat	L4 pyramidal neuron	Unknown
	Ruiz et al. (2003)	Guinea pig	Bouton *en passant*, mossy fiber	Unknown
	Rama et al. (2015a)	Rat	CA3 and L5 pyramidal neuron	Nav deinactivation AP amplitude increase

#### d-ADF *via* Basal Ca^2+^ Accumulation at the Terminal

The first mechanism described for d-ADF is not due to spike shape modulation. A weak opening of synaptic Cavs during the subthreshold depolarization leads to an increase in the basal Ca^2+^ concentration at the terminal, and consequently, an enhancement of synaptic release when the spike invades the presynaptic terminal (Debanne et al., 2013; Rama et al., 2015b; Zbili et al., 2016). This mechanism has been described at the Calyx of Held (Turecek and Trussell, 2001; Awatramani et al., 2005; Hori and Takahashi, 2009), cerebellar molecular layer interneurons (Bouhours et al., 2011; Christie et al., 2011) and at the dendro-dendritic synapses between mitral cells of the olfactory bulb (Fekete et al., 2014). Due to the slow dynamics of Ca^2+^ accumulation, several seconds of depolarization are needed to fully facilitate synaptic transmission. Interestingly, basal Ca^2+^ accumulation induces synaptic release facilitation in two different ways. First, the increase in basal Ca^2+^ concentration directly enhances the release probability, probably *via* the promotion of the vesicles priming and of the coupling between vesicles and Cav channels (Neher and Sakaba, 2008). Second, it can provoke a Ca^2+^-dependent hyperpolarizing shift of Cav channel activation (Borst and Sakmann, 1998; Cuttle et al., 1998), leading to an increase of the spike-evoked Ca^2+^ transient (Hori and Takahashi, 2009; Christie et al., 2011). Importantly, these two effects can occur at the same synapse (Hori and Takahashi, 2009).

#### d-ADF *via* Modulation of Presynaptic Spike Width

The second mechanism underlying d-ADF in mammals is the inactivation of Kv channels during the subthreshold depolarization. This phenomenon provokes broadening of the presynaptic spike (Figures 3Aii,Bii), leading to an increase in the spike-evoked Ca^2+^ transient and an enhancement of synaptic release (Debanne et al., 2013; Rama et al., 2015b; Zbili et al., 2016; Figures 3Ai,Bi). Importantly, the time constant of this type of d-ADF depends on the inactivation time-constant of the Kv involved in the phenomenon. In L5 pyramidal neurons, CA3 pyramidal neurons and CA1 pyramidal neurons, slowly inactivating Kv1 channels are responsible for the d-ADF, and therefore the phenomenon presents a slow time constant (several seconds; Saviane et al., 2003; Shu et al., 2006, 2007; Kole et al., 2007; Sasaki et al., 2011, 2012; Zhu et al., 2011; Kim, 2014; Bialowas et al., 2015). In contrast, in inhibitory interneurons of the cerebellum (stellate cells), d-ADF depends on fast Kv3.4 inactivation and is, therefore, quicker to take place (100 ms of depolarization to have an effect and 1 s for a full increase in synaptic transmission; Rowan and Christie, 2017). Importantly, at invertebrates’ synapses, the d-ADF *via* basal Ca^2+^ accumulation and the d-ADF *via* Kv inactivation are not mutually exclusive and may occur at the same synapses (Bialowas et al., 2015; Rama et al., 2015b).

#### A Peculiar Case: d-ADF at Mossy Fiber Boutons

In granule cells of the Dentate Gyrus, an EPSP can propagate from the dendrites to the presynaptic bouton and increase spike-evoked synaptic transmission at mossy fiber bouton/CA3 synapses (Alle and Geiger, 2006). Surprisingly, this d-ADF seems to be Ca^2+^-independent. In fact, it does not go along with an increase in basal Ca^2+^ concentration or spike-evoked Ca^2+^ transient (Scott et al., 2008), and it is not blocked by application of 10 mM EGTA (Alle and Geiger, 2006) or 1 mM BAPTA (Scott et al., 2008). However, it should be noted that the increase in Ca^2+^ entry can be too subtle to be seen with Ca^2+^ fluorescent indicators. Moreover, at the Calyx of Held, it is necessary to apply 10 mM EGTA + 1 mM BAPTA to efficiently block d-ADF (Hori and Takahashi, 2009). Therefore, the mechanism of d-ADF at this synapse could be Ca^2+^-dependent and needs further studies to be unraveled (Debanne et al., 2013).

### Hyperpolarization-Induced Analog-Digital Facilitation (h-ADF) in Mammalian Brain

Recent studies showed that a presynaptic hyperpolarization before the spike leads to an increase in spike-evoked neurotransmitter release in hippocampal cultures (Thio and Yamada, 2004), L4 pyramidal neurons (Cowan and Stricker, 2004), CA3 pyramidal neurons and L5 pyramidal neurons (Rama et al., 2015a; Figure 3Ci; Table 1). This facilitation ranges between 10% and 100% depending on the studies and the cell type. At CA3/CA3 and L5/L5 synapses, the mechanism underlying h-ADF has been fully described: a presynaptic hyperpolarization results in the recovery from inactivation of presynaptic Nav channels, which provokes an increase in presynaptic spike amplitude (Figure 3Cii), leading to an enhancement of spike-evoked Ca^2+^ entry and synaptic release (Rama et al., 2015a; Figure 3Ci). This phenomenon is likely to occur at hippocampal mossy fiber, in which a somatic hyperpolarization results in an increase of spike-evoked Ca^2+^ transient at small axonal varicosities (Ruiz et al., 2003). Interestingly, due to the fast biophysics of Nav channels, h-ADF is extremely fast and occurs within 15–50 ms of hyperpolarization. Therefore, it can be induced by a unique IPSP preceding the spike. Hence, rebound spiking should have a bigger impact on the postsynaptic cell if h-ADF is present at the synapse. One can think that h-ADF is a widespread phenomenon because it depends on Nav channels which are present in all spiking neuron types. However, it should be noted that a strong Nav channel density should attenuate the impact of their de-inactivation on the spike amplitude, and therefore decrease the h-ADF (Rama et al., 2015a). Moreover, Nav1.6 channels, which are the main subtype in pyramidal cells axons, are strongly inactivated at resting potential (Hu et al., 2009), and therefore are suited to underlie h-ADF. Parvalbumin positive (PV^+^) fast-spiking interneurons contain axonal Nav1.1 channels, a subtype that display less inactivation at resting membrane potential than Nav1.6 (Patel et al., 2015), and a much higher density of axonal Nav channels than pyramidal cells (Hu and Jonas, 2014; Hu et al., 2014). Therefore, one can expect that this type of interneuron lacks spike amplitude modulation and h-ADF.

### Coexistence of d-ADF and h-ADF at the Same Synapses

d-ADF and h-ADF are due to different mechanisms and present different time constants (100 ms to several seconds for d-ADF, 15–50 ms for h-ADF). It has been shown that d-ADF and h-ADF coexist and can be summed at CA3/CA3 synapses (Rama et al., 2015a). In fact, a long depolarization (10 s) followed by a brief hyperpolarization (200 ms) entails a bigger increase in spike-evoked synaptic transmission than a long depolarization or a brief hyperpolarization alone. Why has this coexistence not been shown in early studies in invertebrates? For synapses that display h-ADF but not d-ADF, we can assume a lack of inactivating potassium channels at the synapses studied, which prevents spike broadening by depolarization (giant synapse of the squid and frog neuromuscular junction). In the case of studies that report only d-ADF, it should be noted that the protocol used is not relevant to observe h-ADF. In fact, a brief hyperpolarization (15–200 ms) is needed to unravel h-ADF at synapses that also present d-ADF. A long hyperpolarization (several seconds) can induce de-inactivation of Kv1 channels and spike sharpening, which can thwart h-ADF (Bialowas et al., 2015; Rama et al., 2015a). In the studies made on invertebrates that report d-ADF but no h-ADF, the resting membrane potential was modified for several seconds before spike emission, which may explain why the h-ADF has not been reported. Therefore, additional studies are needed to unravel the degree of coexistence of d-ADF and h-ADF at invertebrates and mammals synapses.

### Physiological Consequences of ADFs

#### Spatial Extent of ADFs

One of the main issues concerning Analog-Digital Facilitations is the spatial extent of these phenomena along the axon. In fact, ADFs are produced by subthreshold modifications of the somatic potential that spreads to the presynaptic terminal and modifies presynaptic spike shape or basal Ca^2+^ (Debanne et al., 2013; Rama et al., 2015b). Therefore, the axonal space constant is a major determinant of the spatial extent of ADF. The axonal space constant varies among neuronal types, depending on the axonal diameter, the density of axonal branching and the axonal membrane resistance (Sasaki et al., 2012).

In CA3 hippocampal neurons, the axonal space constant has been evaluated around 200–500 μm (Sasaki et al., 2012; Bialowas et al., 2015; Rama et al., 2015a). In L5 pyramidal neurons, the value estimated ranges between 500 μm (Shu et al., 2006; Kole et al., 2007) and 1,000 μm (Christie and Jahr, 2009). In CA1 pyramidal neurons, the axonal space constant was found to be around 700 μm (Kim, 2014). Therefore, ADFs seem to be restricted to local brain circuits. For example, d-ADF has been found between CA3 neurons but not at the synapses between CA3 and CA1 neurons (Sasaki et al., 2012). However, several lines of evidence suggest that ADFs could also occur between more distant neurons. First, most of the recordings have been made in young animals (14–30 days after birth), in which the myelin sheet is still in development, and may have underestimated the axonal space constant in myelinated axons, such as CA1 and L5 pyramidal neurons. In fact, cortical myelin has been shown to develop up to 22 months after birth (Hill et al., 2018). Moreover, in myelinated axons of cat motoneurons, the axonal space constant was found to be 1,700 μm (Gogan et al., 1983). Second, neuromodulation can induce direct subthreshold potential modifications of presynaptic terminals by presynaptic receptor activation. Presynaptic glycinergic receptor activation depolarizes the Calyx of Held, leading to an increase in spike-evoked synaptic release (Turecek and Trussell, 2001). Similarly, presynaptic GABA_A_ receptor activation depolarizes the presynaptic terminal, leading to an enhancement of spike-evoked synaptic transmission at mossy fiber giant bouton and Purkinje cells (Ruiz et al., 2010; Zorrilla de San Martin et al., 2017). In contrast, in L5 pyramidal neurons, axonal GABA_A_ receptor activation leads to a hyperpolarization, a decrease of axonal spike width and a decrease of spike-evoked Ca^2+^ entry (Xia et al., 2014). Finally, glutamate released by astrocytes depolarizes terminals in CA3 pyramidal neurons, leading to an increase in spike width and synaptic release (Sasaki et al., 2011). In these latter cases, ADFs are not dependent on subthreshold membrane potential spreading from the soma and could occur even at long range connections.

#### Time Constant of ADFs

ADFs present various time constants which determine their potential roles in network physiology. In fact, in most of the studies, d-ADF needs 100 ms to several seconds of presynaptic depolarization to occur. On the contrary, h-ADF can be produced by fast presynaptic hyperpolarization (15–50 ms; Rama et al., 2015a). This difference is well explained by the underlying mechanism of d-ADF and h-ADF: slow accumulation of basal Ca^2+^ (Bouhours et al., 2011; Christie et al., 2011) or slow Kv inactivation for d-ADF (Shu et al., 2006, 2007; Kole et al., 2007; Bialowas et al., 2015), fast recovery from inactivation of Nav for h-ADF (Rama et al., 2015a; Zbili et al., 2016). Therefore, d-ADF and h-ADF should have different consequences on information transfer in neuronal networks.

#### d-ADF May Maintain Excitatory Synaptic Strength During Cortical Up-States

It has been proposed that d-ADF, due to its slow time-constant, occurs during global network state modifications such as slow-wave sleep associated cortical up and down states (Shu et al., 2006). During an up state, all the neurons of a network will depolarize leading to a global increase in synaptic release through d-ADF. At the same time, the global depolarization should decrease the driving force associated with synaptic excitatory current. In fact, while d-ADF increases synaptic strength by a factor around 1% per mV of presynaptic depolarization (Kole et al., 2007; Bialowas et al., 2015), calculations of driving force for AMPA-driven synaptic current predict a synaptic strength decrease of around 1.5% per mV of postsynaptic depolarization. Therefore, in the case of a globally depolarized neuronal network, d-ADF can be considered as a homeostatic process that maintains an excitatory synaptic strength constant despite the decrease of the driving force.

#### d-ADF May Maintain Excitatory-Inhibitory Balance During Up-States

Interestingly, at connections between L5 pyramidal neurons, a depolarization of the presynaptic pyramidal neuron entails both an increase of the monosynaptic EPSP (i.e., classical d-ADF) and an increase in disynaptic inhibition (Zhu et al., 2011). This depolarization-induced facilitation of disynaptic inhibition results from d-ADF occurring at a synapse between L5 pyramidal neurons and neighboring interneurons, leading to an increase in interneuron firing. This phenomenon has been proposed to be important for the persistence of excitatory-inhibitory balance during up-states where d-ADF may increase excitatory and inhibitory activity at the same time (Zhu et al., 2011).

#### h-ADF May Participate to Network Synchronization

Because of the fast de-inactivation time constant of Nav channels, h-ADF can be induced by a single IPSP preceding the presynaptic spike (Rama et al., 2015a). Therefore, h-ADF modulates synaptic strength on a short time scale and may participate in fast network processing. First, it is well known that hyperpolarization produced by interneurons is able to synchronize the spiking of neighboring pyramidal neurons. Usually, one explains this phenomenon by a transient silencing of the pyramidal cells as a whole, causing the post-hyperpolarization spiking to occur in synchrony. It can also be enhanced by the so-called “rebound spiking,” which is often due to the recovery from inactivation of T-type Ca^2+^ channels and Nav channels during the hyperpolarization. Interestingly, the h-ADF, which increases the inter-pyramidal synaptic strength after a transient hyperpolarization, has been shown to increase the interneuron-driven network synchronization (Rama et al., 2015a). Second, it has been shown that sequences of co-activated pyramidal neurons are often preceded and can be produced by inter-neuronal firing (Sasaki et al., 2014). It would be interesting to examine whether the increase in inter-pyramidal synaptic strength through h-ADF participates in the production of neuronal ensembles by interneurons. Third, h-ADF could be a detection mechanism for unexpected spikes. In fact, when the neuron is hyperpolarized, the spiking probability decreases. However, due to h-ADF, if a spike occurs during a low spiking probability state, the synaptic release is increased, compared to a spike produced at the resting membrane potential. Therefore, h-ADF increases the informational content of unexpected spikes in cortical networks. Finally, it has been proposed that h-ADF participates in the increase of synaptic transmission during silent states of the slow wave sleep, in which cortical neurons can be hyperpolarized by 5–15 mV (Timofeev and Chauvette, 2017).

## Conclusion and Future Directions

We have reviewed evidence showing that the informational content of the spike is dependent on the context of its emission. In fact, the presynaptic spike waveform varies as a function of the neuronal firing rate, the neuro-modulatory state or the subthreshold voltage fluctuations. Moreover, this information is transmitted to postsynaptic neurons by modulation of spike-evoked calcium entry and neurotransmitter release. In this view, the neurotransmission in mammalian CNS needs the occurrence of a digital signal (the spike), whose waveform is modulated by the sub-threshold signal. It has been proposed that synaptic transmission relies on a hybrid between analog and digital signaling, called Analog-Digital synaptic transmission (Clark and Häusser, 2006; Alle and Geiger, 2008). Consequently, the description of APs as purely digital events to study information processing of neuronal networks should be abandoned. In fact, it has been shown that an “AP waveform code” is highly reliable and more informative than a purely digital code (de Polavieja et al., 2005; Juusola et al., 2007). Several issues have to be solved to unravel the physiological implications of context-dependent spike waveform modulation. First, ADFs should be tested at various stages of neuronal development. One could expect that context-dependent modulation of spike waveform is larger in developing networks, which present a lower density of voltage-gated channels and therefore a less stable spike shape. Second, d-ADF and h-ADF have to be studied in adult networks, in which the myelination process is completed, to confirm the spatial extent of these phenomena. In fact, two published studies report paired recordings of thalamo-cortical connections (Bruno and Sakmann, 2006; Hu and Agmon, 2016), making it possible to test if ADFs occur at long range connections when the axon is myelinated. Third, it would be interesting to observe the extent of ADFs in interneurons. In fact, due to their high voltage-gated channel density, it seems that fast-spiking cortical interneurons do not present spike waveform modulation in function of the membrane potential (Tateno and Robinson, 2006; Goldberg et al., 2008). In contrast, cerebellar stellate cells present depolarization-induced spike broadening and an increase in synaptic release (Rowan and Christie, 2017). Therefore, GABAergic transmission may undergo ADF, depending on the type of interneuron studied. Finally, the spike waveform effects may not be restricted to synaptic release and may affect dendritic computing. In fact, it has been proposed that a spike broadening causes a shunting of incoming PSPs and a decrease in their summation, promoting a low firing rate (Hausser et al., 2001; Juusola et al., 2007). Interestingly, in L5 pyramidal neurons, depolarization-induced spike broadening has been shown to have a shorter time-constant in the soma than in the axon (Shu et al., 2007), due to local differences in potassium current expression (fast inactivating I_A_ current in the somato-dendritic compartment and slow inactivating I_D_ current in the axon). Moreover, in contrast with the axonal spike broadening, it seems that the dendritic spike broadening reduces the Ca^2+^ entry through the decrease of the driving force promoting the Ca^2+^ tail current in granule cells of the dentate gyrus (Brunner and Szabadics, 2016). Therefore, it is likely that subthreshold depolarization has different effects on somato-dendritic and axonal computing. The pursuit of the investigation on neuronal computation *via* spike waveform will certainly unravel the physiological importance of this phenomenon on networks dynamics.

## Author Contributions

MZ and DD wrote the manuscript and built the figures.

## Conflict of Interest Statement

The authors declare that the research was conducted in the absence of any commercial or financial relationships that could be construed as a potential conflict of interest.
